# +mRNA expression of LRRC55 protein (leucine-rich repeat-containing protein 55) in the adult mouse brain

**DOI:** 10.1371/journal.pone.0191749

**Published:** 2018-01-25

**Authors:** Ying-Ying Zhang, Xue Han, Ye Liu, Jian Chen, Lei Hua, Qian Ma, Yang-Yu-Xin Huang, Qiong-Yao Tang, Zhe Zhang

**Affiliations:** 1 School of Anesthesiology, Xuzhou Medical University, Xuzhou, Jiangsu Province, China; 2 Jiangsu Province Key Laboratory of Anesthesiology, Xuzhou Medical University, Xuzhou, Jiangsu Province, China; 3 Jiangsu Province Key Laboratory of Anesthesia and Analgesia Application Technology, Xuzhou Medical University, Xuzhou, Jiangsu Province, China; Virginia Commonwealth University, UNITED STATES

## Abstract

LRRC55 (leucine-rich repeat-containing protein 55) protein is an auxiliary γ subunit of BK (Big conductance potassium channel) channels, which leftward shifts GVs of BK channels around 50 mV in the absence of cytosolic Ca^2+^. LRRC55 protein is also the only γ subunit of BK channels that is expressed in mammalian nervous system. However, the expression pattern of LRRC55 gene in adult mammalian brain remains elusive. In this study, we investigated the distribution of LRRC55 mRNA in the adult mouse brain by using in situ hybridization. We found that LRRC55 mRNA is richly expressed in the adult mouse medial habenula nucleus (MHb), cerebellum and pons. However, the potential role of LRRC55 in MHb and cerebellum could be different based on the function of BK channels in these brain regions.

## Introduction

LRRC (leucine-rich repeat-containing) proteins form a superfamily that contains several hundred member proteins. Each member protein possesses two or more leucine rich repeated motifs (LRR). LRR motif is composed of 20–29 amino acid residues rich in leucine and other aliphatic amino acids. The function of LRR motifs is suggested as providing versatile framework for forming protein-protein interactions [1]. Furthermore, LRRC proteins have diverse functions including antibacterial reaction, maintenance of normal cardiac function, regulation of trafficking of membrane receptors and regulation of activity of ion channels, etc [2,3,4,5]. Among LRRC proteins, LRRC55, LRRC38, LRRC52 and LRRC26 are identified as auxiliary proteins of BK (Big conductance of K^+^) channels, which can leftward shift GVs of Slo1 channels from several tens to 120 mV [6].

BK channels comprise a potassium channel family that includes three members: Slo1, Slo2 and Slo3 channels [7]. Each channel consists of four pore-forming α subunits that co-assemble to form a tetramer channel with a pore domain between S5 and S6 transmembrane segments. Slo1 channels are Ca^2+^ activated potassium channels while Slo2 channels are gated by sodium [8,9]. Slo3 channels are specifically expressed in testis and activated by alkalization [10,11,12]. Slo1 channels are also regulated by β1 to β4 subunits, which confer slow activation time course and/or inactivation character to Slo1 channels [13,14,15]. The function of Slo1 channels and their β subunits in epilepsy, hypertension and regulation of adrenal medullary chromaffin cells had also been widely studied [16,17,18]. Over-activation mutants of Slo1 and Slo2.2 channel lead to epilepsy whereas Slo1 KO mice demonstrate hypertension [19,20,21]. However, until recently, LRRC proteins had been identified as γ subunits of BK channels, which constitute a new family of BK channels auxiliary proteins. LRRC26 protein is the first γ subunit identified from non-excitable cancer cells [5]. In mouse, LRRC26 protein is at least expressed in 3 tissues: lacrimal gland, parotid gland, and colon [22]. LRRC38 protein is also mainly expressed in secretory glands [6,22]. LRRC52 protein is mainly expressed in testis, within which it can interact with Slo3 channels [23]. LRRC55 protein seems the only γ subunit of BK channels expressed in nervous system that may alter biophysical property and confer novel function to LRRC55-complexed Slo1 channels in LRRC55 protein expressed brain nuclei. However, the distribution of LRRC55 in the adult mouse brain has not been investigated. In this study, we examined the expression pattern of LRRC55 mRNA in the whole mouse brain by using in situ hybridization. We found that LRRC55 mRNA is richly expressed in the medial habenula nucleus (MHb), cerebellum and pons of the adult mouse. This result suggests potential special functions of LRRC55-complexed Slo1 channels in these brain regions.

## Material and methods

### Animals and surgical procedure

All experimental procedures were performed according to institutional safety and ethnical using animal rules that are agreed by IACUC (Institutional Animal Care and Use Committee) of Xuzhou Medical University. Experimental mouse strain is the C57BL/6J mouse strain. Male mice at (8-12weeks of age) was anesthetized by 4% chloral hydrate intraperitoneal injection at 0.5mg/Kg body weight. Total 6 mice were used in this experiment. The mice were provided by SPF (specific pathogen free) animal house of Xuzhou Medical University. Mice were housed in 12h daily dark-light cycle in cages under 4 mice/cage condition in core facility. A completed ARRIVE guidelines checklist is included in S1 Checklist.

### Molecular biology and in situ probe synthesis

The N-terminal 292bp DNA fragment of the mouse LRRC55 gene (Genebank accession number NM_001033346) was synthesized by the Genewiz Company. The gene fragment was cloned into pBbluescriptSK vector between EcoRI and BamHI site. After cleaving vector by BamHI restriction endonuclease, the antisense RNA probe of LRRC55 was *in vitro* transcribed with T7 transcriptase by following procedures of Digoxigenin (DIG) labeled RNA probe synthesizing kit. The sense control cRNA probe for LRRC55 mRNA was *in vitro* transcribed by T3 transcriptase after EcoRI restriction endonuclease digestion.

### In situ hybridization

C57BL/6J mice [male, at 8–12 weeks of age (wk)] were deeply anesthetized and transcardially perfused with 4% paraformaldehyde (PFA) in 0.1 M phosphate buffer (PB) solution (pH 7.4). Then the mice were decapitated, and their brains were quickly dissected out and soaked in 30% sucrose. After the brain precipitated in 30% sucrose, the brain was successively cut as 20 μm thick coronal sections. Sections were collected on Superfrost/Plus slides purchased from Fisher Company and air-dried one hour at room temperature, then stored at -80°C or were used for in situ hybridization immediately. In situ hybridization was performed according to Anderson lab protocol as previously described [24].

## Results

### Localization of LRRC55 transcripts in the adult mouse forebrain and midbrain by in situ hybridization

**Fig 1:**
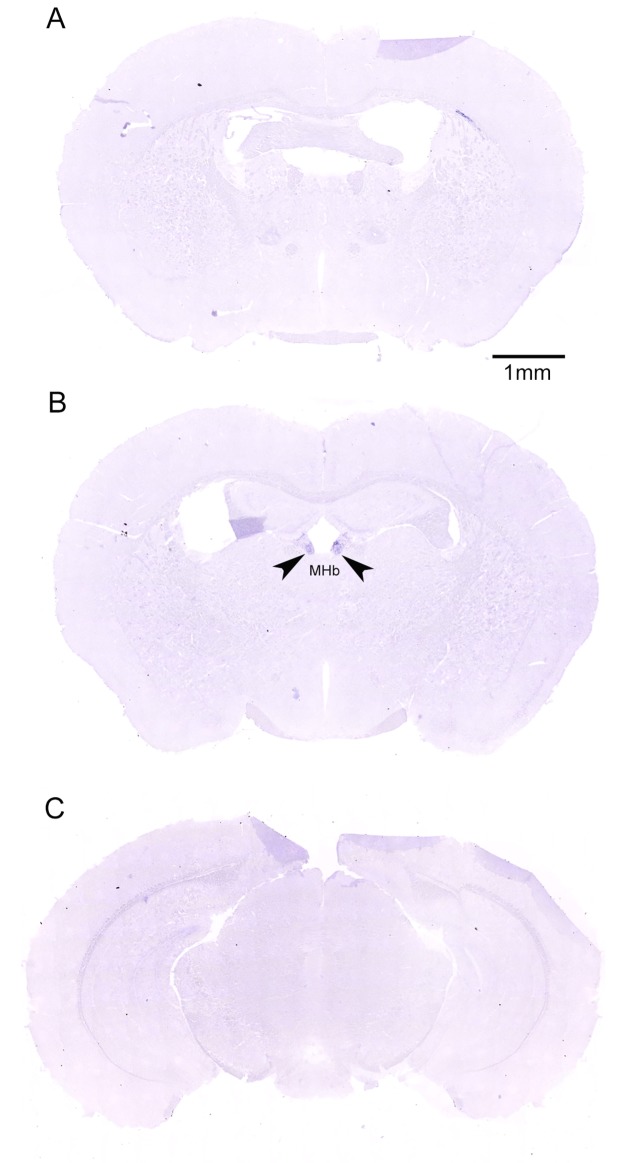
Digoxigenin labeled in situ hybridization detected specific expression of LRRC55 mRNA in the adult mouse medial habenula nucleus (MHb). Coronal sections at each level of the brain were hybridized with either antisense probe or sense probe for LRRC55 mRNA. Sections hybridized with antisense probe along the rostral-caudal axis were shown. Sections that are rostral to **(A)** and caudal to **(C)** the medial habenula nucleaus (MHb) failed to show any LRRC55 mRNA positive signal staining. But sections at the level of the medial habenula nucleus (MHb) showed LRRC55 mRNA is only expressed in the adult mouse MHb **(B)**.

**Fig 2:**
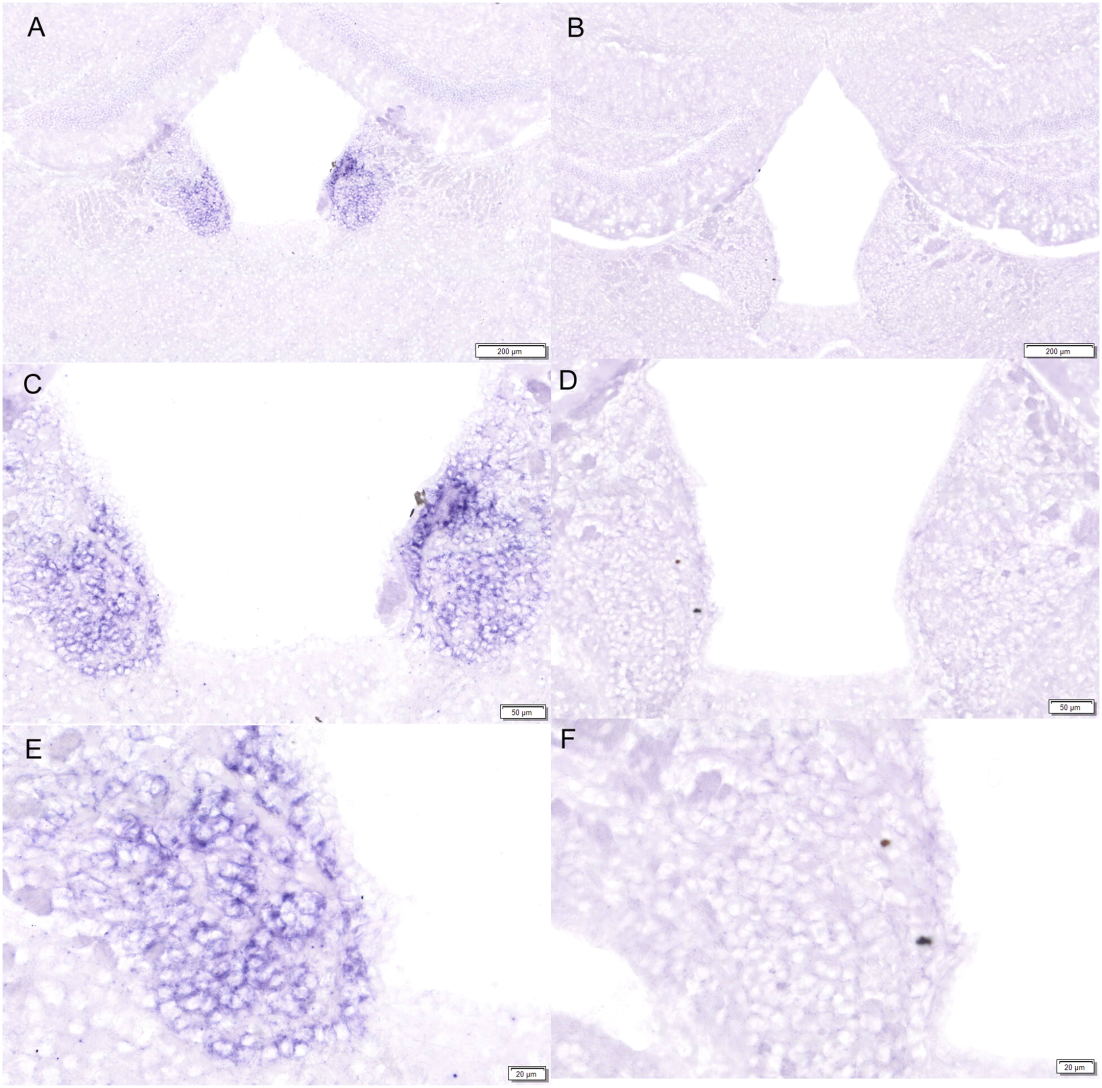
High magnification photographs showed LRRC55 mRNA is located in the cytoplasm of nerve cells in the medial habenula nucleus (MHb). Purple signals labelled by antisense probe for LRRC55 mRNA were observed in nerve cells in the adult mouse MHb (4-fold magnification) **(A)**. No positive signal was detected by sense probe for LRRC55 mRNA in the MHb **(B)**. Higher magnification photographs showed strong positive signals (purple staining) were located in the cytoplasm of nerve cells in the MHb **(C, E.** 10-fold and 40-fold magnification, respectively**)**. No positive hybridization signal appeared in the adult mouse MHb on sections hybridized by sense probe for LRRC55 mRNA. (**D, F.** 10-fold and 40-fold magnification, respectively).

Expression of the LRRC55 gene in the adult mouse brain was examined by Digoxigenin (DIG) labeled in situ hybridization. Antisense RNA probe and sense RNA control probe of the LRRC55 gene were used to screen series of coronal sections along the rostral-caudal axis throughout the whole brain. As shown in (Fig 1A), no positive signal was detected on brain sections by antisense LRRC55 RNA probe until sections reached the level of the medial habenula nucleus (MHb). On sections at the level of the medial habenula nucleus, positive hybridization signals detected by anti-sense LRRC55 RNA probe were only observed in the medial habenula nucleus (MHb) (Fig 1B). The positive signal was also not observed on sections at levels caudal to the MHb until sections cross levels of the whole hypothalamus (Fig 1C). These results indicate that LRRC55 mRNA is specifically expressed in the MHb in the adult mouse forebrain and midbrain. Higher magnification (4-fold to 40-fold) photographs of coronal sections at the level of MHb showed that purple positive hybridization signals were located in the cytoplasm of nerve cells in the MHb (Fig 2A, 2C and 2E), but there was no positive purple signal detected by the sense LRRC55 RNA probe in the MHb (Fig 2B, 2D and 2F). Although there were some swallow purple staining signals in the hippocampus, similar signals were also detected by the sense probe in the hippocampus on control sections. Thus, we consider those signals are background staining (Fig 2C and 2D).

### Localization of LRRC55 transcripts in the adult mouse hindbrain by in situ hybridization

**Fig 3:**
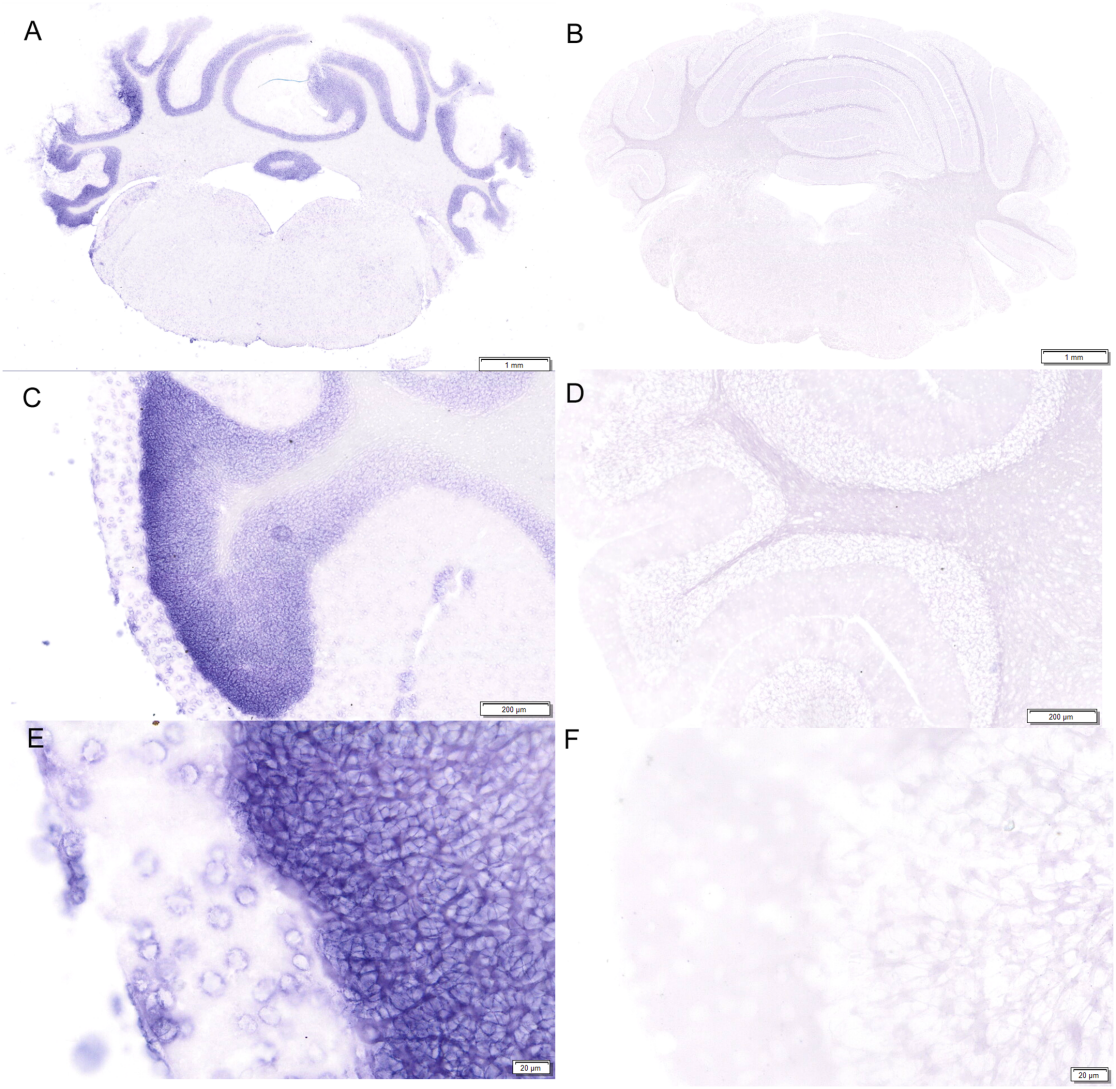
LRRC55 mRNA expression is rich in the cerebellum and pons of adult mice. Whole section photograph showed positive signals of LRRC55 mRNA expression detected by DIG labeled Anti-sense probe were in the granule layer and molecular cell layer in the adult mouse cerebellum **(A)**. No positive signal was detected by sense probe for LRRC55 mRNA in the adult mouse cerebellum and pons **(B)**. Higher magnification photos revealed that LRRC55 mRNA are widely expressed in the cytoplasm of nerve cells in the granule and molecular layer in the adult mouse cerebellum (**C, E.** 10-fold and 40-fold magnification, respectively). No positive signal was detected by sense probe for LRRC mRNA in the adult mouse cerebellum (**D, F.** 10-fold and 40-fold magnification, respectively).

**Fig 4:**
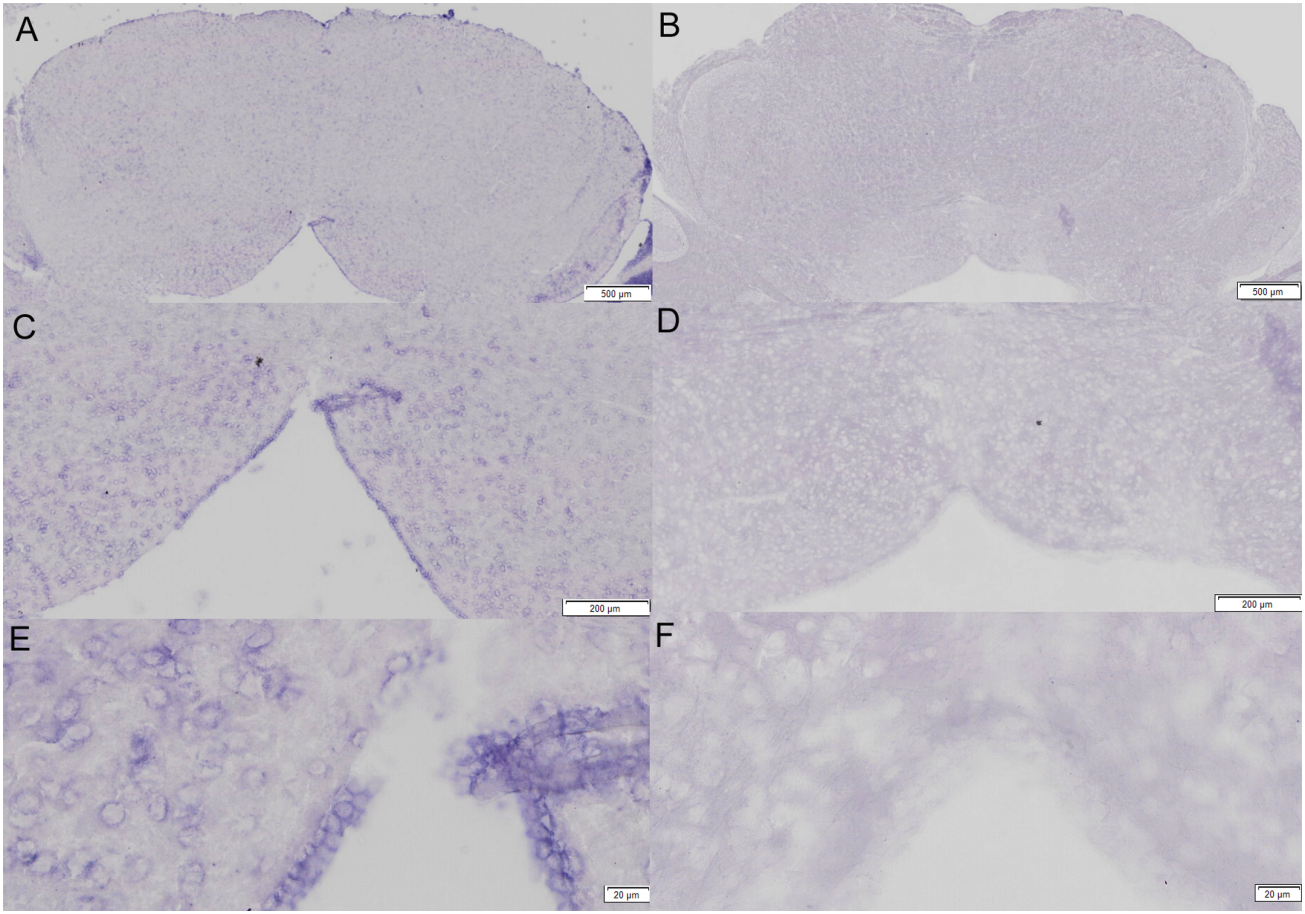
Different magnification photographs showed LRRC55 mRNA is richly expressed in cytoplasm of nerve cells in the adult mouse pons. Anti-sense probe detected that LRRC55 mRNA is widely expressed in the adult mouse pons (**A, C, E**. 1, 10 and 40-fold magnification, respectively). Sense probe for LRRC55 mRNA failed to detect any positive hybridization signal in the adult mouse pons (**B, D, F.** 1, 10 and 40-fold magnification, respectively). Bars: as labelled.

LRRC55 mRNA expression was also detected in the cerebellum and the pons (Fig 3A and 3B). 10-fold to 40-fold magnification photographs showed that strong purple signals of LRRC55 mRNA expression were detected in the granule cell and molecular cell layers of the cerebellum by anti-sense LRRC55 probe. (Fig 3C and 3E). In the meantime, we failed to detect any positive signal of LRRC55 mRNA expression in the cerebellum by the sense control probe (Fig 3D and 3F). In addition, LRRC55 mRNA expression in the pons is also rich. 4-fold to 40-fold magnification photographs showed wide strong positive signals for mRNA expression of LRRC55 in the pons detected by antisense probe (Fig 4A, 4C and 4E) while no positive signal for LRRC55 mRNA expression was shown in the pons on control sections screened by sense probe (Fig 4B, 4D and 4F).

In all, our results show LRRC55 mRNA is selectively expressed in the adult mouse MHb, cerebellum and pons. These results suggest Slo1 channels in these brain regions possess special biophysical properties conferred by LRRC55 protein. As a result, they probably perform special function in these brain regions.

## Discussion

In the present study, non-radioactive in situ hybridization was used to thoroughly investigate the distribution of LRRC55 transcripts in the adult mouse brain. Our data indicate that LRRC55 mRNA is dominantly expressed in the medial habenula nucleus (MHb), cerebellum and pons in the adult mouse, which suggests the unique biophysical property and function of Slo1 channels in these nuclei. These findings are partially inconsistent with previous report that LRRC55 mRNA is expressed in mitral cells in the olfactory bulb and 4a/6a layers in cortex in mice [25]. However, the previous report used brain sections in mice age E15 and P0, which cannot be used to account for expression pattern of LRRC55 mRNA in the adult mouse brain. Thus, LRRC55 mRNA distribution in the mouse brain is probably regulated by the age of the mouse. The consistent part is that both studies found that LRRC55 mRNA expression is specifically rich in the medial habenula nucleus (MHb). In addition, our study also reports that LRRC55 mRNA is richly expressed in the adult mouse cerebellum and the pons, which was not reported by previous study. Thus, our findings provide new insights on LRRC55 mRNA expression pattern in adult mammalian brain.

Although several kinds of LRRC55 antibodies are commercial available, the specificity of these antibodies has not been confirmed by Western blot. Thus, we have not considered to use them to examine LRRC55 protein expression pattern in the mouse brain. But we believe the LRRC55 mRNA distribution in brain largely reflects the expression pattern of LRRC protein in brain. In general, despite expression levels of transcripts do not always reflect those of related proteins, it is conceivable that rich mRNA can be efficiently translated into proteins.

The mSlo1 channels are ubiquitously expressed in nervous system. But LRRC55 subunits mRNA expression is restricted in the MHb in the mouse forebrain and midbrain, which offers special biophysical property to the Slo1 channels in medial habenula nucleus. Furthermore, studies show that the MHb plays an important role in stress, depression, memory, motor related behavior and nicotine withdrawal [26,27]. However, recent studies show that although BK channel current can be recorded in MHb but it does not regulate activity of nicotinic acetylcholine receptors (nAchR), which suggests no involvement of LRRC55-complexed Slo1 channels in nicotine withdrawal function. But it is possible that the BK channel and LRRC55 protein are involved in depression and stress. In addition, the rich LRRC55 mRNA expression in the cerebellum and pons also suggests LRRC55 modified Slo1 channels may be involved in motor related function because Slo1 channels are also richly expressed cerebellum and pons [28,29,30]. Previous study also suggests that BK channels are involved in cerebellar ataxia [31]. Taken together, we tentatively suggest that LRRC55-mslo1 complex may be involved in motor learning related functions. Thus, the special regulation of LRRC55 on BK channels may play different roles in MHb and cerebellum respectively. As a consequence, our results provide clues for further studying the role of LRRC55 and Slo1 channel played in nervous system.

## Supporting information

S1 Checklist Completed “the ARRIVE guidelines checklist” for reporting animal data in this manuscript. (DOCX)
