# Correction: +mRNA expression of LRRC55 protein (leucine-rich repeat-containing protein 55) in the adult mouse brain

**DOI:** 10.1371/journal.pone.0197531

**Published:** 2018-05-10

**Authors:** Ying-Ying Zhang, Xue Han, Ye Liu, Jian Chen, Lei Hua, Qian Ma, Yang-Yu-Xin Huang, Qiong-Yao Tang, Zhe Zhang

There are errors in the Funding section. The correct funding information is as follows: This work was supported by The Important Project of Natural Science in Colleges and Universities in Jiangsu Province to Z.Z. (14KJA320002), Jiangsu specially appointed professorship to Z.Z. and Q.-Y.T., Natural Science Foundation of China (NSFC) grant to Z.Z. (81471314, 81671090), Natural Science Foundation of Jiangsu Province to Z.Z (BK20151170). Xuzhou Science and Technology Program (KC16SG251) to Z.Z. NSFC grant to Q.-Y.T. (31671212). Training programs of innovation and Entrepreneurship for undergraduates of Jiangsu Province in 2014 (201410313065X). We also appreciated the grant support from the Priority Academic Program Development of Jiangsu Higher Education Institutions and from the Jiangsu Provincial Special Program of Medical Science (BL2014029). The funders had no role in study design, data collection and analysis, decision to publish, or preparation of the manuscript.
