# Supplementary material for: +mRNA expression of LRRC55 protein (leucine-rich repeat-containing protein 55) in the adult mouse brain
Source: PLoS One. 2018 Jan 25;13(1):e0191749. doi: 10.1371/journal.pone.0191749 (PMC5784982; doi:10.1371/journal.pone.0191749)

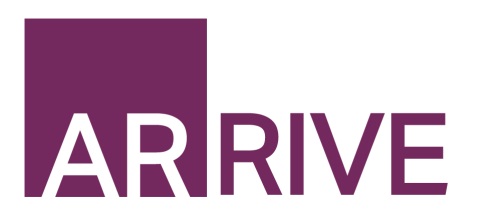


The ARRIVE Guidelines Checklist

Animal Research: Reporting In Vivo Experiments

**mRNA expression of LRRC55 protein (leucine-rich repeat-containing protein 55) in the adult mouse brain**

Ying-Ying Zhang^1^, Xue Han^1^, Ye Liu^2,3^, Jian Chen^1^, Lei Hua^1^, Qian Ma^1^, Yang-Yu-Xin Huang^1^, Qiong-Yao Tang^1,2,3^, Zhe Zhang^1,2,3,*^

*^1^ School of Anesthesiology, Xuzhou Medical University, Xuzhou, Jiangsu Province 221004, China*

*^2^ Jiangsu Province Key Laboratory of Anesthesiology, Xuzhou Medical University, Xuzhou, Jiangsu Province 221004, China*

*^3^ Jiangsu Province Key Laboratory of Anesthesia and Analgesia Application Technology, Xuzhou Medical University, Xuzhou, Jiangsu Province*

*221004, China*

**Correspondence: zhangzhe70@xzhmu.edu.cn*

|  | | ITEM | RECOMMENDATION | Section/ Paragraph |
| --- | --- | --- | --- | --- |
| 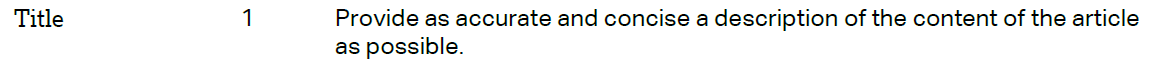 | | | Title, First line |  |
| 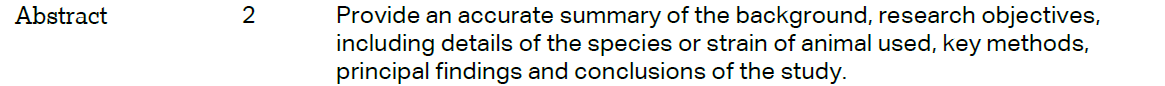 | | | Abstract, paragraph 1 |  |
| INTRODUCTION | | |  |  |
| 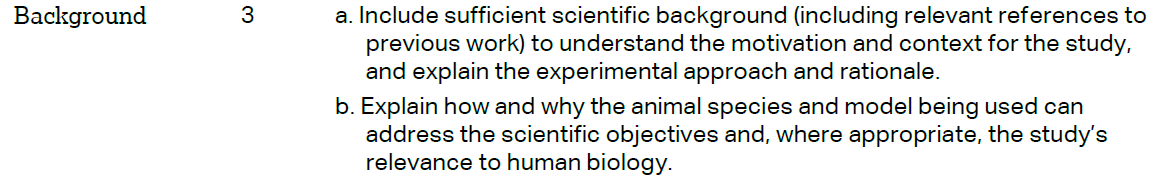 | | | Introduction, paragraph 1-2 |  |
| 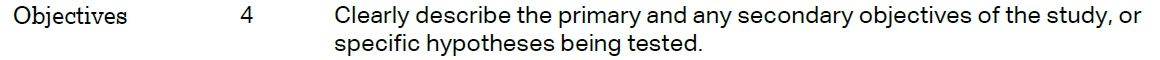 | | | Introduction, paragraph 2, last 5 lines. |  |
| METHODS | | |  |  |
| 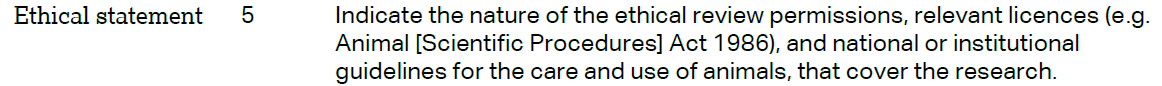 | | | Material and Method,  Animals and surgical procedures, paragraph 1 |  |
| 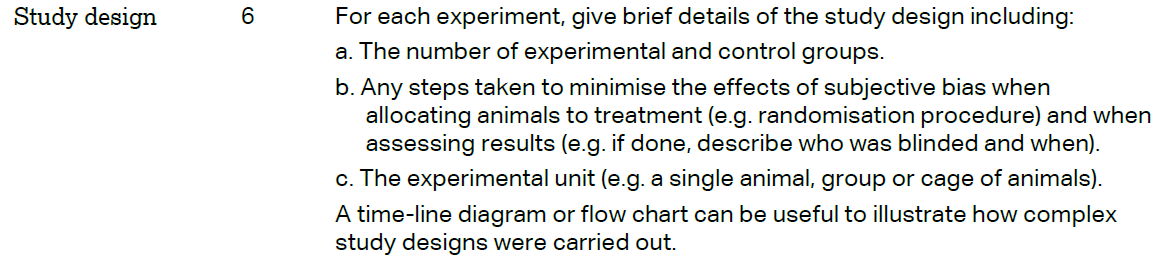 | | | Material and Methods,  Molecular biology and in situ hybridization. |  |
| 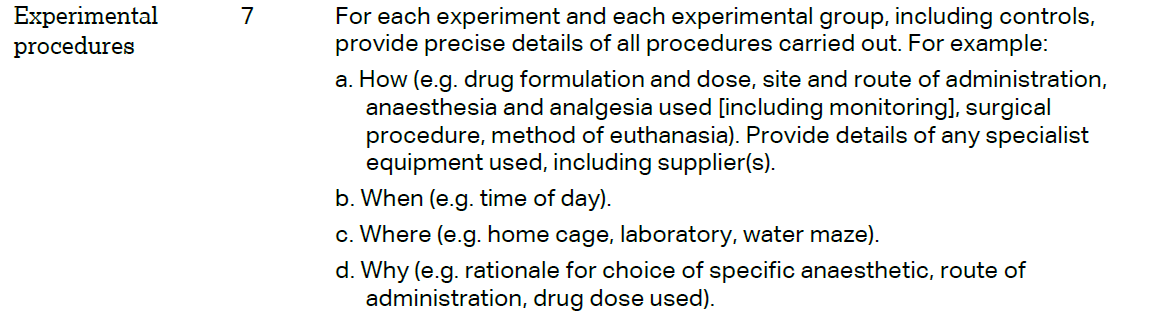 | | | Material and Method,  In situ hybridizatio-n.  parargraph 1 |  |
| 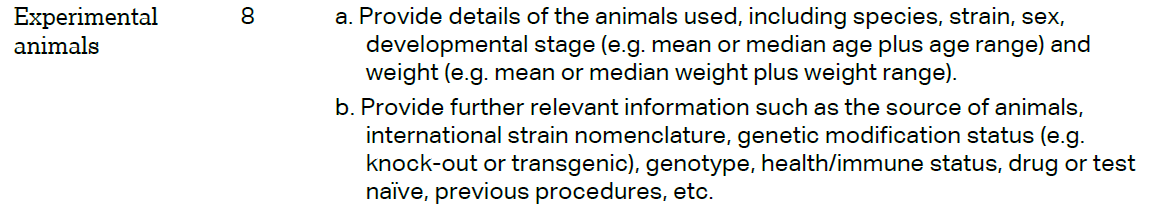 | | | Material and method,  Animal and surgical procedures  Paragraph1 |  |

The ARRIVE guidelines. Originally published in *PLoS Biology*, June 2010^1^

| 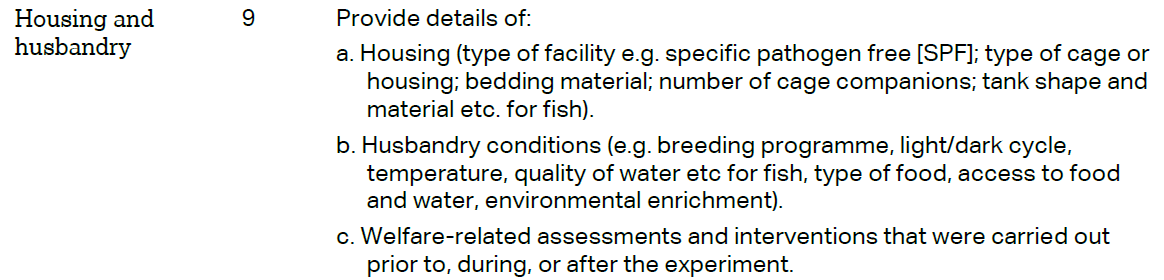 | Material and method，animal and surgery procedures, paragraph 1 | |
| --- | --- | --- |
| 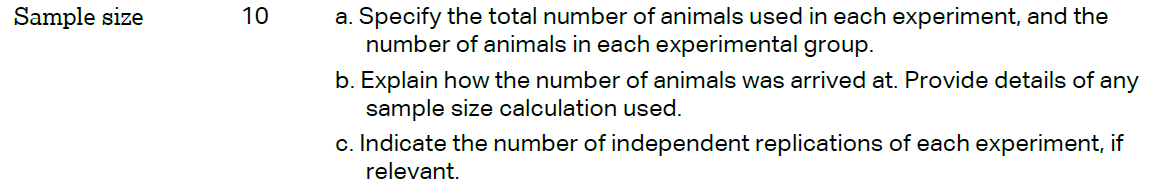 | Material and method, animal and surgery procedures, paragraph 1 | |
| 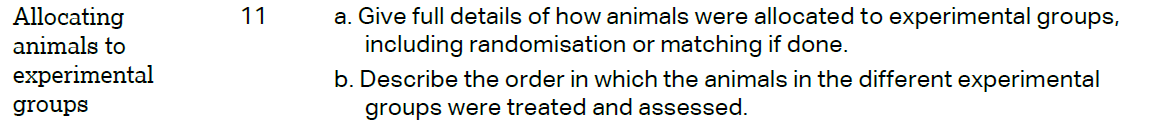 | Material and method, animal and surgery procedures, paragraph 1 | |
| 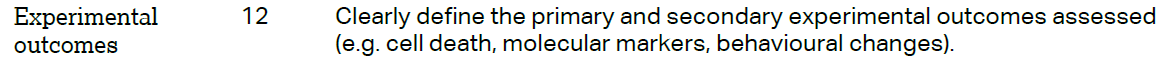 | Results,  All paragraph. | |
| 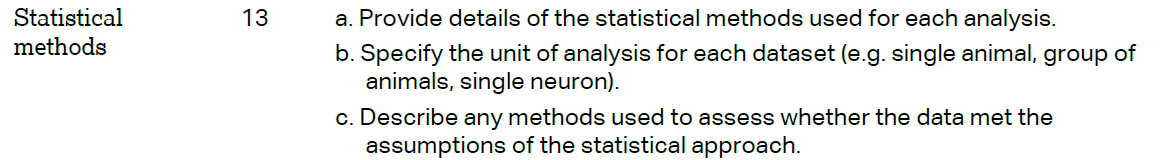 | No statistical methods had been used | |
| RESULTS |  | |
| 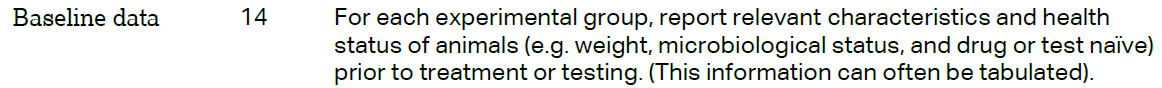 | Material and methods, animal and surgery procedures, paragraph 1 | |
| 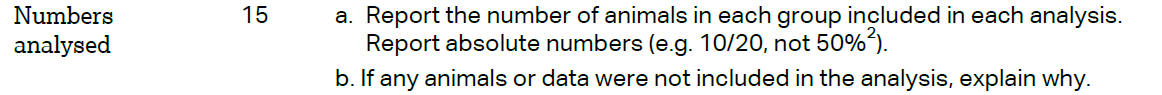 | Material and methods, animal and surgery procedures | |
| 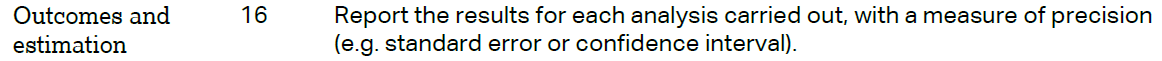 | Results, all paragraphs. No statistical method was used. | |
| 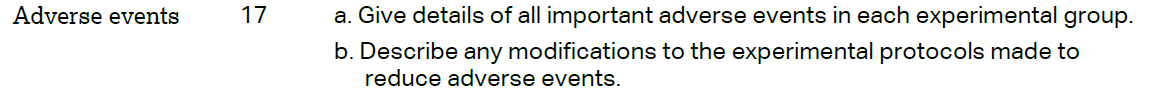 | Not applicable. Results show no adverse effect | |
| DISCUSSION |  | |
| 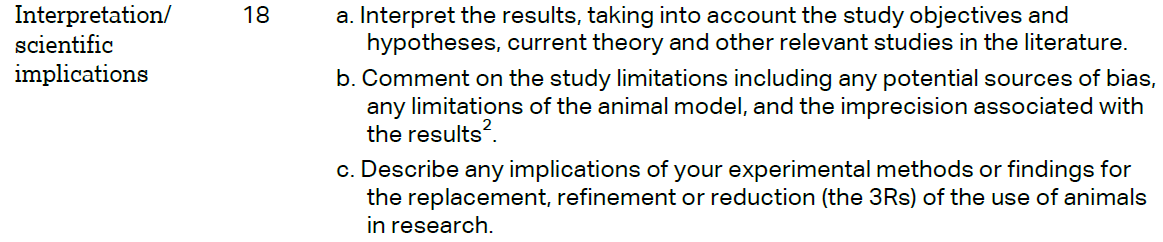 | Result，all paragraphs  Discussion,  Paragraph 1. | |
| 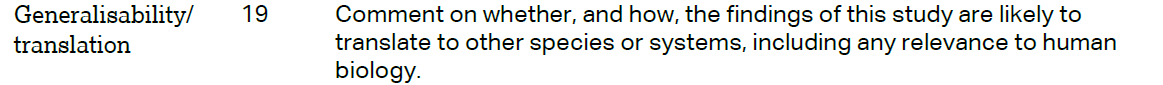 | Not applicable.  We did not report any translation study | |
| 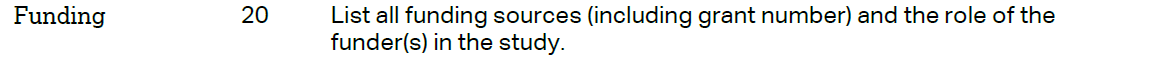 | | Acknowledgement, paragraph 1 |


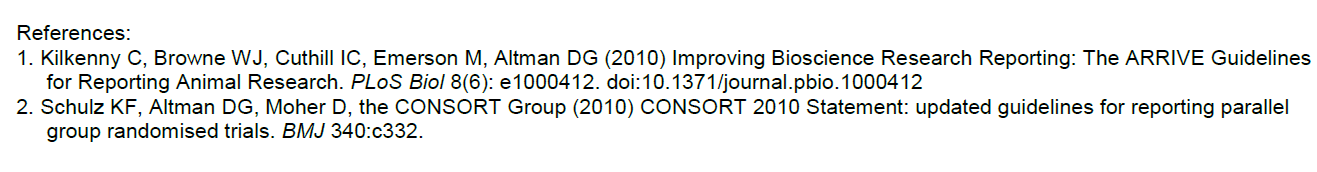

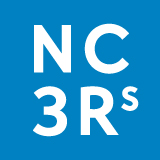

Supplement: S1 Checklist — (DOCX) [file pone.0191749.s001.docx]
